# Technology in Rehabilitative Interventions for Children: Challenges and Opportunities

**DOI:** 10.3390/children9050598

**Published:** 2022-04-22

**Authors:** Daniela Traficante, Alessandro Antonietti

**Affiliations:** Department of Psychology, Catholic University of the Sacred Heart, 20123 Milan, Italy; alessandro.antonietti@unicatt.it

Technology innovation has been leading to the development of an increasing number of applications that aim to support the rehabilitation of cognitive functions. What are the pros and cons of the use of such applications in training programs addressing children with neurodevelopmental disorders? The papers included in this Special Issue offer the opportunity to explore this topic from different perspectives as they are focused not only on the efficacy of interventions mediated by technology [1,2,3] but also on the impact that the use of technology in telerehabilitation, in particular during the COVID-19 pandemic, has had on wellbeing experience of children with Specific Learning Disorders and Cerebral Palsy [4]. Moreover, due to the relevance of the use of technology in the treatment of Specific Learning Disorders, Lorusso et al. [5] carried out a Delphi study to propose a set of best practices in order to guide the use of technology to realize training programs and to support learning processes in school activities.

The first question addressed by the Special Issue is the efficacy of the use of technology in the treatment of neurodevelopmental disorders. Lino et al.’s narrative review [1] presents an overview on the use of serious games using Virtual Reality (VR) and Augmented Reality (AR) to treat Developmental Coordination Disorder (DCD) in childhood. The authors argued that the “Internal Modeling Deficit” (IMD) characterizes the compromised motor ability of children with DCD. More precisely, the internal models of motor control are supposed to stem from two components: Mental Imagery (MI) and Action Observation (AO). Even though digital technology can offer the opportunity to support both these components, through the visualization and reproduction of motor patterns and strategies in children with DCD, few studies assessed the effectiveness of training based on VR/AR technologies. To date, there is preliminary evidence that such technologies can be beneficial due to the high level of children’s engagement and enjoyment in attending treatment sessions with VR/AR, but further research is needed to obtain reliable data on their efficacy.

As for Specific Learning Disorders, Lorusso et al. [2] present their experience from the application of an automated training program, Tachidino, aimed at the treatment of reading and writing disorders. The software is based on the Visual Attention Training (AVG) and the Visual-Hemisphere-Specific Stimulation (VHSS) approach, which is grounded on Bakker’s Balance Model. It allows therapists to tailor the duration and the flexibility of intervention in order to support the child’s engagement and reading fluency. The assessment of the efficacy of the training program showed encouraging results, irrespective of the age of the children and the severity of the disease. Hence, such a training program can be considered a useful tool to promote reading ability in children with Dyslexia.

Serious videogames, such as the activities included in Tachidino, are worth considering because of their interactive modality. In addition, they fit for the implementation of telerehabilitation, which played a major role in supporting the wellbeing and the treatment of children with neurodevelopmental disorders during the COVID-19 pandemic. In fact, in that condition, technology offered therapists the opportunity to assure the rehabilitation program would continue. The unusual situation caused by the pandemic led researchers and practitioners to face a new question, i.e., the influence of the modality of the training implementation (online vs. in presence) on treatment efficacy. In order to give some clues on this issue, Cancer et al.’s study [3] compared the effects of Rhythmic Reading Training (RRT), realized under the direct supervision of the practitioner present, and the effects observed when the training was applied online under the remote control of the therapist to remediate reading deficits in children with Dyslexia. The results showed that there was not any difference between the two modalities. Therefore, this study can be considered a piece of evidence to support the use of telemedicine and telerehabilitation in the context of interventions addressed to pediatric populations.

The relevance of telerehabilitation in supporting the wellbeing experience in children with neurodevelopmental disorders is well documented by the contribution of Sarti et al. [4], who assessed the experience of children with Specific Learning Disorders and Cerebral Palsy during the pandemic period. The authors compared children who were involved in online treatment to children who did not have the opportunity to undertake telerehabilitation by analyzing a group of typically developing children (control group) as well. Children who were engaged in telerehabilitation showed a higher level of involvement in learning activities, with a higher level of perceived social support and respect, than the control group.

As the role of technology and telerehabilitation has been rising during the last decade and is likely to increase in the future, it might be important to identify the best practices that can provide professionals with suggestions and share experience among practitioners in order to offer effective support to the greatest number of children in any condition of implementation of the treatment. The paper by Lorusso et al. [5] presents a synthesis of a Delphy study conducted among Italian professionals, aiming to endorse some statements on the use of new technologies applied in the treatments of Dyslexia, which will be included within a European project on the use of VR and AR in learning to read. Overall, the respondents showed a positive attitude towards the use of Information and Communication Technology in rehabilitation programs and appreciated, in particular, the application of VR in training programs aimed at the automation of grapheme-to-phoneme conversion rules. 

We currently have a large number of technological tools that can be employed in remediation interventions addressed to neurodevelopmental disorders at our disposal and the trend is constantly increasing, with new devices being and their implementation becoming widespread. What is crucial now is understanding the directions to be followed in order to orientate the efforts to improve the quality of rehabilitation thanks to technology towards relevant goals. To do so, the comprehension of the true added value of technological instruments is important. Technology is seductive because novelty generates expectations and enthusiasm, but what is new is not always better as well. A critical approach is needed to distinguish appearance from actual benefits. On the one hand, technology allows practitioners to optimize procedures, which indeed can be implemented through traditional means. In this case, the advantage produced by technology consists of saving time, improving precision, reducing costs, shortening the training periods of therapists, and so forth. On the other hand, technology allows professionals to do things that cannot be carried out with traditional tools, such as reaching new populations, involving parents in the treatment, combining different media and formats, and so on. The first kind of benefits must not be neglected, but obviously, the second kind is more intriguing and this seems to be the most interesting direction. In this perspective, the challenge is identifying the processes that underlie the application of technological devices: What changes across the rehabilitation treatment when certain tools are used? Do changes concern attitudes, motivation, feelings, cognition, communication, or social relations? Additionally, more specifically, which attitudes, motives, feelings, cognitive, communicative, and social processes are involved? We hope the Special Issue can provide readers some insights into this topic and suggest some possible future steps for research and intervention.
